# An Efficient Method Based on Framelets for Solving Fractional Volterra Integral Equations

**DOI:** 10.3390/e22080824

**Published:** 2020-07-28

**Authors:** Mutaz Mohammad, Alexander Trounev, Carlo Cattani

**Affiliations:** 1 Department of Mathematics & Statistics, Zayed University, Abu Dhabi 144543, UAE; 2 Department of Computer Technology and Systems, Kuban State Agrarian University, Krasnodar 350044, Russia; trounev.a@edu.kubsau.ru; 3 Engineering School (DEIM), Tuscia University, 01100 Viterbo, Italy; Cattani@tu.it

**Keywords:** framelets, numerical solution, fractional calculus, generalization of Unequal Error Protection (UEP), wavelets, harmonic numerical analysis, volterra integral equations

## Abstract

This paper is devoted to shedding some light on the advantages of using tight frame systems for solving some types of fractional Volterra integral equations (FVIEs) involved by the Caputo fractional order derivative. A tight frame or simply framelet, is a generalization of an orthonormal basis. A lot of applications are modeled by non-negative functions; taking this into account in this paper, we consider framelet systems generated using some refinable non-negative functions, namely, B-splines. The FVIEs we considered were reduced to a set of linear system of equations and were solved numerically based on a collocation discretization technique. We present many important examples of FVIEs for which accurate and efficient numerical solutions have been accomplished and the numerical results converge very rapidly to the exact ones.

## 1. Introduction

Fractional calculus is an old topic; it was started from some fractional order derivative questions raised by Leibniz in 1695 and Euler 1730 but a yet novel one. It has been developed through extensive work to date. Many mathematicians have been involved and contributed dramatically to the field, such as Fourier, Laplace, Riesz and many more. Most recently, numerous scientists provided new definitions of fractional order derivatives and integrals that opened a new era in the history of fractional derivatives, such as the Atangana–Baleanu fractional integral [1], the Caputo fractional derivative [2] and the Caputo–Fabrizio fractional derivative [3]. There is a series of new lines of research that is devoted to fractional calculus and its applications in many disciplines, such as physics, engineering and modeling [4,5,6,7,8,9,10,11,12,13,14,15,16,17,18,19,20,21,22,23,24,25,26,27,28,29,30,31,32,33,34,35,36,37,38].

In the literature, there are plenty of contributions on the use of wavelets and their generalizations to model and solve several problems of differential and integral equations of different types and applications in pure mathematics, engineering and physics; see, for example [39,40,41,42,43,44,45,46,47,48,49,50,51,52,53,54,55]. In this paper, we use framelets with three generators generated via set of B-splines in order to solve fractional Volterra integral equations (FVIEs). Usually, it is difficult and sometimes impossible to find exact solutions for such types of integral equations. Therefore, developing numerical algorithms aimed to find a numerical approximation is essential.

In this paper, we consider the following form of fractional Volterra integral equation (FVIE)

(1) $$\begin{array}{c} D^{\lambda }u\left(x\right)=a\left(x\right)u\left(x\right)+b\left(x\right)+\int_{0}^{x}K(x,t)u(t)dt,\;x\in \left[0,1\right],\;\lambda >0, \end{array}$$

with the following initial conditions (ICs)

(2) $$\begin{array}{c} u^{\left(p\right)}\left(0\right)=d_{p},\;\;p=0,1,2,\cdots ,m-1,\;and\;\lambda \in \left(n,n+1\right],n\in N, \end{array}$$

where $D^{\lambda }u$ is the known Caputo fractional order derivative given by

$$D^{\lambda }u\left(x\right)=\frac{1}{\Gamma (n-\lambda )}\int_{0}^{x}{\left(x-t\right)}^{n-\lambda -1}\frac{d^{n}u\left(t\right)}{dt}dt.$$

The purpose here is to provide an approximate solution of the fractioal Volterra initial value problem (FVIVP) given in Equations (1) and (2) in the form of the truncated expansions of a framelet system, where a set of functions $\left\{u_{j},j=1,\cdots ,\infty \right\}$ is called a framelet for $L^{2}\left(R\right)$ if there exists a positive number c,C such that the inequality

(3) $${c∥v∥}^{2}\leq \sum_{j=1}^{\infty }{\left|\langle v,u_{j}\rangle \right|}^{2}\leq C{∥v∥}^{2},$$

holds for any function $v\in L^{2}\left(R\right).$

Note that according to the inequality (3), for a function $g\in L^{2}\left(R\right)$ it is obvious to obtain the following associated framelet representation

(4) $$g=\sum_{j\in Z}\langle g,u_{j}\rangle u_{j}.$$

The framelets are constructed using B-spline functions. The B-splines $B_{M}$ of order *M* are defined recursively by the following equation

$$B_{M}\left(x\right)=\int_{0}^{1}B_{M-1}\left(x-t\right)dt,\;M=1,2,\cdots ,$$

where $B_{1}\left(x\right)$ is the indicator function over [0,1).

B-splines are non-negative refinable functions in the sense that

$${\hat{B}}_{M}\left(\xi \right)=\hat{a}\left(\xi /2\right)\hat{\phi }\left(\xi /2\right),$$

where

$$\hat{a}\left(\xi \right)=2^{-n}{\left(1+e^{-i\xi }\right)}^{n}p\left(\xi \right),$$

such that p(ξ) is a polynomial of trigonometric functions with p(0)=1, and $\hat{a}$ is 2π-periodic function in the frequency domain and called the low mask of $B_{M}$.

The framelet system X(Ξ) is constructed via the oblique extension principle (OEP) [39] and has the form

$$X\left(\Xi \right)==\left\{u_{\ell ,j,k}=2^{j/2}u\left(2^{j}x-k\right):\;\ell =1,\cdots ,r;j,k\;\mathrm{are}\mathrm{integers}\right\}$$

and satisfies the following equations

$$\sum_{\ell =0}^{r}{\left|{\hat{a}}_{\ell }\left(\xi \right)\right|}^{2}=1\;\;and\;\;\sum_{\ell =0}^{r}{\hat{a}}_{\ell }\left(\xi \right){\hat{a}}_{\ell }\left(\xi +\pi \right)=0,$$

where ${\hat{a}}_{0},{\hat{a}}_{\ell },\ell =1,\cdots ,r,$ are the low and high masks of the $u=B_{M},$ respectively.

The representation in Equation (4) is truncated by the series $P_{n}$, such that

(5) $$P_{n}g=\sum_{\ell =1}^{r}\sum_{j=-n}^{n}\sum_{k\in Z}\langle g,u_{\ell ,j,k}\rangle u_{\ell ,j,k}.$$

Let us present some examples of framelet systems.

**Example 1.**  *Consider the refinable function, $B_{2}\left(x\right)$. Then, based on the OEP presented in [39] we are able to construct the following framelets explicitly,*

$$\begin{array}{ccc} {\hat{\psi }}_{1}\left(\omega \right) & = & \frac{2\sqrt{2}e^{\frac{i\omega }{2}}}{\pi \omega^{2}}+\frac{e^{-i\omega }}{4\sqrt{2}\pi \omega^{2}}-\frac{9e^{i\omega }}{4\sqrt{2}\pi \omega^{2}}+\frac{e^{2i\omega }}{4\sqrt{2}\pi \omega^{2}}-\frac{9}{4\sqrt{2}\pi \omega^{2}},\\ {\hat{\psi }}_{2}\left(\omega \right) & = & -\frac{\sqrt{2}e^{\frac{i\omega }{2}}}{\pi \omega^{2}}-\frac{\sqrt{2}e^{\frac{3i\omega }{2}}}{\pi \omega^{2}}+\frac{3e^{i\omega }}{\sqrt{2}\pi \omega^{2}}+\frac{e^{2i\omega }}{2\sqrt{2}\pi \omega^{2}}+\frac{1}{2\sqrt{2}\pi \omega^{2}},\\ {\hat{\psi }}_{3}\left(\omega \right) & = & -\frac{3e^{i\omega }}{4\sqrt{2}\pi \omega^{2}}+\frac{3e^{2i\omega }}{4\sqrt{2}\pi \omega^{2}}-\frac{e^{3i\omega }}{4\sqrt{2}\pi \omega^{2}}+\frac{1}{4\sqrt{2}\pi \omega^{2}}. \end{array}$$

**Figure 1:**
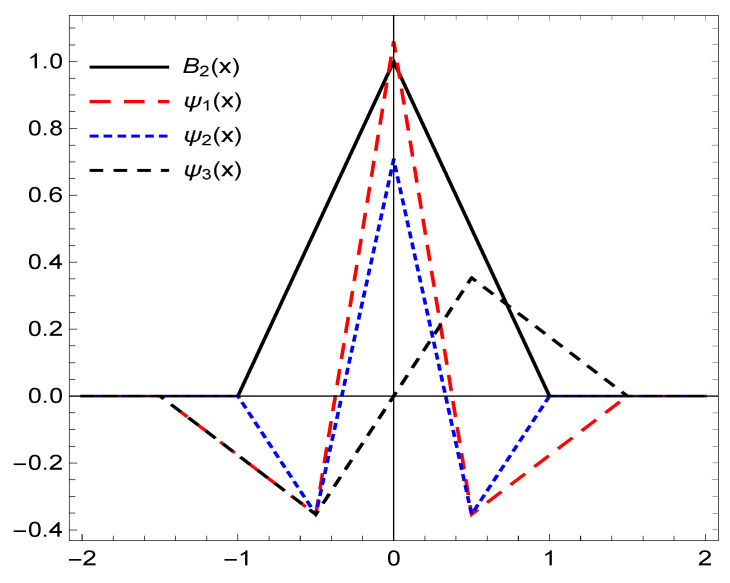
The graphs of the functions in $X(\Xi_{1})$ for Example 1.

*Then, the system $X(\Xi_{1})$ where $\Xi_{1}=\left\{\psi_{1},\psi_{2},\psi_{3}\right\}$ forms a framelet system for $\in L^{2}\left(R\right)$. The graphs of the framelets are plotted in Figure 1.*

**Example 2.**  *Consider the refinable function, $B_{4}\left(x\right)$. Then, again based on the OEP we have*

$$\begin{array}{ccc} {\hat{\psi }}_{1}\left(\omega \right) & = & 0.0241443e^{-5i\omega }{\left(-1+e^{i\omega /2}\right)}^{8}\left(8e^{i\omega /2}+e^{i\omega }+1\right)\omega^{-4},\\ {\hat{\psi }}_{2}\left(\omega \right) & = & 0.132663{\left(-1+e^{-i\omega /2}\right)}^{8}\left(8e^{-i\omega /2}+28.52e^{-i\omega }+8e^{-3i\omega /2}+e^{-2i\omega }+1\right)\omega^{-4},\\ {\hat{\psi }}_{3}\left(\omega \right) & = & 0.143418{\left(-1+e^{-i\omega /2}\right)}^{8}\left(26.4789\left(e^{-i\omega }+e^{-2i\omega }\right)+8e^{-i\omega /2}+43.8315e^{-3i\omega /2}+8e^{-5i\omega /2}+e^{-3i\omega }+1\right)\omega^{-4}. \end{array}$$

**Figure 2:**
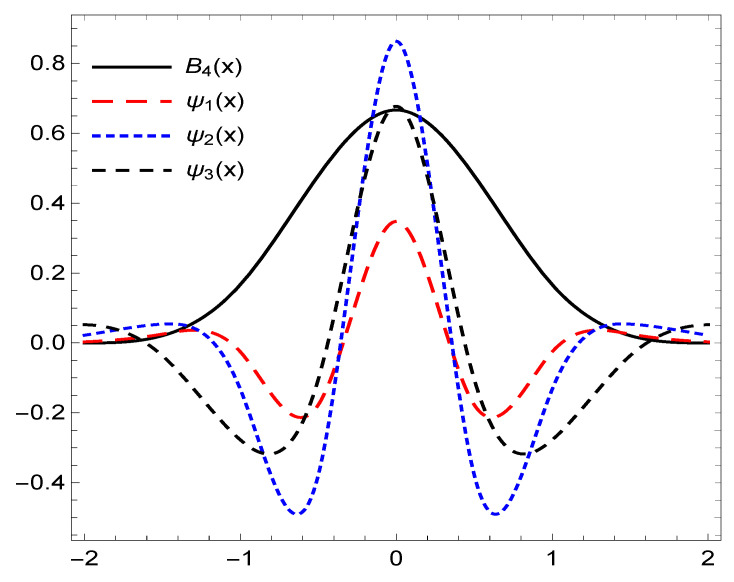
The graphs of the functions in $X(\Xi_{2})$ for Example 2.

*Then, the system $X(\Xi_{2})$ where $\Xi_{2}=\left\{\psi_{1},\psi_{2},\psi_{3}\right\}$ forms a framelet system for $\in L^{2}\left(R\right)$. The graphs of the framelets are plotted in Figure 2.*

## 2. Matrix Formulation Using Framelets

In this section, we provide the general framework of the aforementioned numerical scheme based on the collocation discretization of the domain. We also provide two results related to the existence and uniqueness of the solution.

Consider the FVIE defined in Equation (1). Based on the truncated expansion obtained in Equation (5), we have

$$\begin{array}{c} D^{\lambda }u\left(x\right)=a\left(x\right)I^{n}\left(P_{m}u\left(x\right)\right)+b\left(x\right)+\int_{0}^{x}K\left(x,t\right)I^{n}\left(P_{m}u\left(t\right)\right)dt, \end{array}$$

where the *n*th derivative is approximated by the truncated framelet expansion as follows:

$$u^{\left(n\right)}=P_{m}u,$$

and $I^{\lambda }$ is the Riemann–Liouville fractional-integral operator defined by

$$I^{\lambda }\left(g\right)\left(x\right)=\frac{1}{\Gamma (\lambda )}\int_{0}^{x}\frac{g(t)}{{\left(x-t\right)}^{1-\lambda }}dt.$$

Therefore, using the Caputo derivative, we then get

(6) $$\begin{array}{c} \frac{1}{\Gamma (n-\lambda )}\int_{0}^{x}\frac{P_{m}u\left(t\right)}{{\left(x-t\right)}^{n+1-\lambda }}dt=b\left(x\right)+\frac{1}{\Gamma (\lambda )}\int_{0}^{x}\frac{a\left(x\right)P_{m}u\left(t\right)}{{\left(x-t\right)}^{1-\lambda }}dt+\frac{1}{\Gamma (\lambda )}\int_{0}^{x}\int_{0}^{t}\frac{K\left(x,y\right)P_{m}u\left(y\right)}{{\left(x-y\right)}^{1-\lambda }}dydt. \end{array}$$

With a little algebra, Equation (6) can be simplified to the following

$$\begin{array}{ccc} \frac{1}{\Gamma (n-\lambda )}\int_{0}^{x}\frac{u^{\left(n\right)}\left(t\right)}{{\left(x-t\right)}^{n+1-\lambda }}dt & - & \frac{1}{\Gamma (\lambda )}\int_{0}^{x}\sum_{\ell =1}^{r}\sum_{j\leq m,k}\frac{c_{j,k}^{\ell }\left(u\right)a\left(x\right)\psi_{\ell ,j,k}\left(t\right)}{{\left(x-t\right)}^{1-\lambda }}dt+\\ & & -\frac{1}{\Gamma (\lambda )}\int_{0}^{x}\int_{0}^{t}\sum_{\ell =1}^{r}\sum_{j\leq m,k}\sum_{\ell =1}^{r}\sum_{j\leq m,k}\frac{c_{j,k}^{\ell }\left(u\right)K\left(x,y\right)\psi_{\ell ,j,k}\left(y\right)}{{\left(x-y\right)}^{1-n}}dydt=b\left(x\right). \end{array}$$

Now, based on a dyadic discretization points of the domain of the framelet system being used, say, $\left\{\xi_{q},q\in \Delta \right\}$, and by plugging these point into the equations above, we have

$$\begin{array}{ccc} \frac{1}{\Gamma (n-\lambda )}\int_{0}^{\xi_{q}}\frac{u^{\left(n\right)}\left(t\right)}{{\left(\xi_{q}-t\right)}^{n+1-\lambda }}dt & - & \frac{1}{(n-1)!}\int_{0}^{\xi_{q}}\int_{0}^{t}\sum_{\ell =1}^{r}\sum_{j\leq m,k}\frac{c_{j,k}^{\ell }\left(u\right)a\left(\xi_{q}\right)\psi_{\ell ,j,k}\left(s\right)}{{\left(\xi_{q}-s\right)}^{1-n}}dsdt+\\ & & -\int_{0}^{\xi_{q}}\int_{0}^{t}\sum_{\ell =1}^{r}\sum_{j\leq m,k}\sum_{\ell =1}^{r}\sum_{j\leq m,k}\frac{c_{j,k}^{\ell }\left(u\right)K\left(\xi_{q},y\right)\psi_{\ell ,j,k}\left(y\right)}{{\left(\xi_{q}-y\right)}^{1-n}}dydt=b\left(\xi_{q}\right). \end{array}$$

The above equation yields a system of equations that can be easily solved to obtain the unknown coefficients $c_{j,k}^{\ell }\left(u\right)$ in order to get the approximate solution of order *m*.

We now provide two main results with regard to the existence and uniqueness of the FVIVP defined in Equations (1) and (2).

**Theorem 1**  (Existence). *Assume that a, b and K are continuous functions on [0,1]. Then there exists a real-valued function u defined on [0,ξ] solving the FVIVP given in Equations (1) and (2) such that*

$$\xi_{1}=\min\left\{1,{\left(\frac{\epsilon_{1}\Gamma \left(\lambda +1\right)}{{∥a∥}_{\infty }{∥u∥}_{\infty }+{∥b∥}_{\infty }}\right)}^{1/\lambda }\right\},$$

*and*

$$\xi_{2}=\min\left\{1,{\left(\frac{\epsilon_{2}\Gamma \left(\lambda +1\right)}{{∥K∥}_{\infty }{∥u∥}_{\infty }}\right)}^{1/(\lambda +1)}\right\},$$

*where $\xi =\min\left\{\xi_{1},\xi_{2}\right\},\epsilon_{1}+\epsilon_{2}<\epsilon ,$ and $\epsilon_{1},\epsilon_{2}>0$.*

**Proof.**  Apply the Riemann–Liouville integral operator of both sides of Equation (1), and using the ICs we have,

$$L\left(u\right)\left(x\right)=u\left(x\right)=d_{p}+\frac{1}{\Gamma (\lambda )}\left(\int_{0}^{x}\frac{a(t)u(t)+b(t)}{{\left(x-t\right)}^{1-\lambda }}dt+\int_{0}^{x}\int_{0}^{s}\frac{K(s,t)u(t)}{{\left(s-t\right)}^{1-\lambda }}dtds\right).$$

The idea is to show that L is a self mapping operator on the non-empty set Y where

$$Y=\left\{u\in C\left[0,\xi \right]:∥u-d_{p}{∥}_{\infty }<\epsilon \right\},$$

and has a fixed point in Y. Hence

$$\begin{array}{ccc} \left|L\left(u\right)\left(x\right)-d_{p}\right| & = & \frac{1}{\Gamma (\lambda )}\left|\int_{0}^{x}\frac{a(t)u(t)+b(t)}{{\left(x-t\right)}^{1-\lambda }}dt+\int_{0}^{x}\int_{0}^{s}\frac{K(s,t)u(t)}{{\left(s-t\right)}^{1-\lambda }}dtds\right|\\ \left|L\left(u\right)\left(x\right)-d_{p}\right| & \leq & \frac{1}{\Gamma (\lambda )}\left|\int_{0}^{x}\frac{a(t)u(t)+b(t)}{{\left(x-t\right)}^{1-\lambda }}dt\right|+\frac{1}{\Gamma \left(\lambda \right)}\left|\int_{0}^{x}\int_{0}^{s}\frac{K(s,t)u(t)}{{\left(s-t\right)}^{1-\lambda }}dtds\right|\\ & \leq & \frac{1}{\Gamma (\lambda +1)}\left({∥a∥}_{\infty }{∥u∥}_{\infty }+{∥b∥}_{\infty }\right)x^{\lambda }+\frac{{∥K∥}_{\infty }{∥u∥}_{\infty }}{\Gamma (\lambda +2)}x^{\lambda +1}\\ & \leq & \epsilon_{1}+\epsilon_{2}\\ & < & \epsilon . \end{array}$$

Which means L(u)∈Υ is a self mapping function and this completing the proof. □

**Theorem 2**  (Uniqueness). *Assume that a, b and K are continuous functions on [0,1]. Let $C_{a},C_{b}$ and $C_{K}$ are upper bounds for a, b and K, respectively. Then, the FVIVP defined in Equations (1) and (2) has a unique solution if*

$$0<\frac{\left(\lambda +1\right)C_{a}+C_{K}}{\Gamma (\lambda +2)}<1.$$

**Proof.**  Assume that the FVIVP has two solutions $u_{1}$ and $u_{2}$. Then, we have

$$D^{\lambda }u_{1}=a\left(x\right)u_{1}\left(x\right)+b\left(x\right)+\int_{0}^{x}K\left(x,t\right)u_{1}\left(t\right)dt,$$

and

$$D^{\lambda }u_{2}=a\left(x\right)u_{2}\left(x\right)+b\left(x\right)+\int_{0}^{x}K\left(x,t\right)u_{2}\left(t\right)dt.$$

By taking the Riemann–Liouville integral, we get

$$u_{1}\left(x\right)-d_{p}=\frac{1}{\Gamma \left(\lambda \right)}\int_{0}^{x}\frac{a\left(x\right)u_{1}\left(x\right)+b\left(x\right)}{{\left(x-t\right)}^{1-\lambda }}dt+\frac{1}{\Gamma \left(\lambda \right)}\int_{0}^{x}\int_{0}^{s}\frac{K\left(s,t\right)u_{1}\left(t\right)}{{\left(s-t\right)}^{1-\lambda }}dtds,$$

and

$$u_{2}\left(x\right)-d_{p}=\frac{1}{\Gamma \left(\lambda \right)}\int_{0}^{x}\frac{a\left(x\right)u_{2}\left(x\right)+b\left(x\right)}{{\left(x-t\right)}^{1-\lambda }}dt+\frac{1}{\Gamma \left(\lambda \right)}\int_{0}^{x}\int_{0}^{s}\frac{K\left(s,t\right)u_{2}\left(t\right)}{{\left(s-t\right)}^{1-\lambda }}dtds.$$

Note that

$$\begin{array}{ccc} \left|u_{2}-u_{1}\right| & \leq & \frac{1}{\Gamma \left(\lambda \right)}\left|\int_{0}^{x}\frac{a\left(t\right)(u_{2}\left(t\right)-u_{1}\left(t\right))}{{\left(x-t\right)}^{\lambda }}dt+\int_{0}^{x}\int_{0}^{s}\frac{K\left(s,t\right)(u_{2}\left(t\right)-u_{1}\left(t\right))}{{\left(s-t\right)}^{1-\lambda }}dtds\right|\\ & \leq & \frac{1}{\Gamma \left(\lambda \right)}\left|\int_{0}^{x}\frac{\left|a\left(t\right)(u_{2}\left(t\right)-u_{1}\left(t\right))\right|}{{\left(x-t\right)}^{\lambda }}dt+\int_{0}^{x}\int_{0}^{s}\frac{\left|K\left(s,t\right)(u_{2}\left(t\right)-u_{1}\left(t\right))\right|}{{\left(s-t\right)}^{1-\lambda }}dtds\right|\\ & \leq & \frac{C_{a}}{\lambda \Gamma \left(\lambda \right)}|u_{2}-u_{1}{\left|\;|x\right|}^{\lambda }+\frac{C_{K}}{\left(\lambda +1)\lambda \Gamma (\lambda \right)}{\left|x\right|}^{\lambda +1}\\ & \leq & \frac{\Gamma \left(\lambda +1\right)C_{a}+C_{K}}{\Gamma (\lambda +2)}\left|u_{2}-u_{1}\right|. \end{array}$$

Therefore, as

$$|u_{2}-u_{1}|\left(\eta -1\right)\geq 0,$$

where $0<\eta =\frac{\Gamma \left(\lambda +1\right)C_{a}+C_{K}}{\Gamma (\lambda +2)}<1$, the result is concluded. □

## 3. Numerical Performance

In this section we consider some examples to test the proposed algorithm. The absolute errors is given by

$$E_{m}u=|u-I^{n}\left(P_{m}u\right)|,\lambda \leq n.$$

**Example 3.**  *Consider the following FVIE,*

(7) $$\left\{\begin{array}{c} D^{1/2}u\left(x\right){-}_{0}^{x}u(t)dt=\frac{8x^{3/2}}{3\sqrt{\pi }}-\frac{x^{3}}{3}-x^{2},\\ u(0)=0,\\ u^{\prime }\left(0\right)=0. \end{array}\right.$$

*Note that, Equation (7) can be rewritten as*

$$\left\{\begin{array}{c} {\frac{1}{\Gamma \left(1/2\right)}}_{0}^{x}\frac{u^{\prime }\left(t\right)}{{\left(x-t\right)}^{1/2}}dt{-}_{0}^{x}u(t)dt=\frac{8x^{3/2}}{3\sqrt{\pi }}-\frac{x^{3}}{3}-x^{2},\\ u(0)=0,\\ u^{\prime }\left(0\right)=0. \end{array}\right.$$

**Figure 3:**
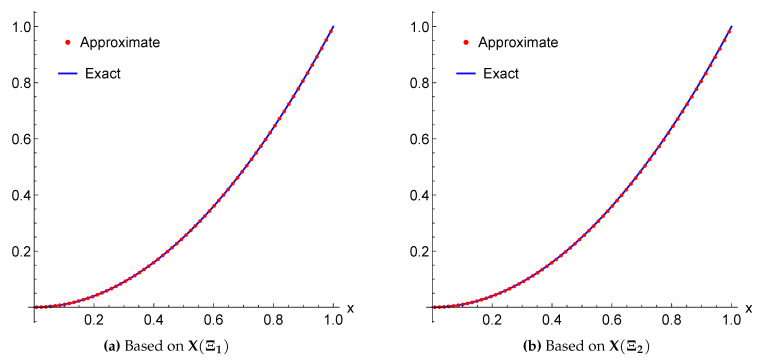
A Comparison between the exact and approximate solutions of Example 3 for m=3 using the framelet systems $X(\Xi_{1})$ and $X(\Xi_{2})$.

**Figure 4:**
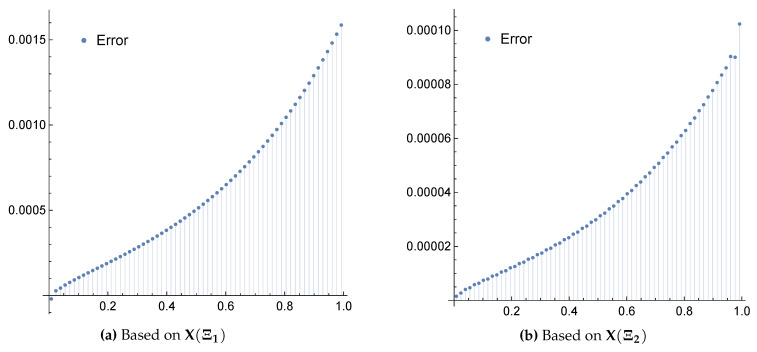
Error plots for Example 3 when m=4 and using the framelet systems $X(\Xi_{1})$ and $X(\Xi_{2})$.

*The exact solution for this equation is $u\left(x\right)=x^{2}.$ Applying the above scheme yields the numerical results presented in Table 1 and Table 2 and the graphical illustration for the comparison of exact, approximate and error results in Figure 3 and Figure 4.*

**Example 4.**  *Consider the following FVIE.*

(8) $$\left\{\begin{array}{c} D^{3/4}u\left(x\right){-}_{0}^{x}x\sin(t)u(t)dt=\frac{1}{\Gamma (5/4)}x^{1/4}+\left(x\sin\left(x+\frac{\pi }{2}\right)-\sin\left(x\right)\right)u\left(x\right),\\ u(0)=0. \end{array}\right.$$

*Note that Equation (8) can be reduced to*

$$\left\{\begin{array}{c} {\frac{1}{\Gamma \left(1/4\right)}}_{0}^{x}\frac{u^{\prime }\left(t\right)}{{\left(x-t\right)}^{3/4}}dt{-}_{0}^{x}x\sin(t)u(t)dt=\frac{1}{\Gamma (5/4)}x^{1/4}+\left(x\sin\left(x+\frac{\pi }{2}\right)-\sin\left(x\right)\right)u\left(x\right).\\ u(0)=0. \end{array}\right.$$

**Figure 5:**
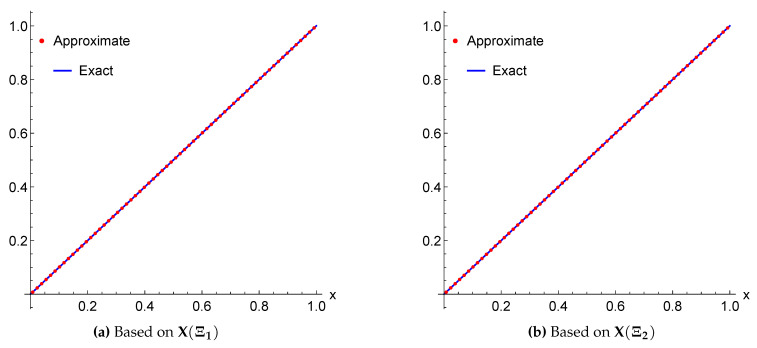
A Comparison between the exact and approximate solutions of Example 4 for m=3 using the framelet systems $X(\Xi_{1})$ and $X(\Xi_{2})$.

**Figure 6:**
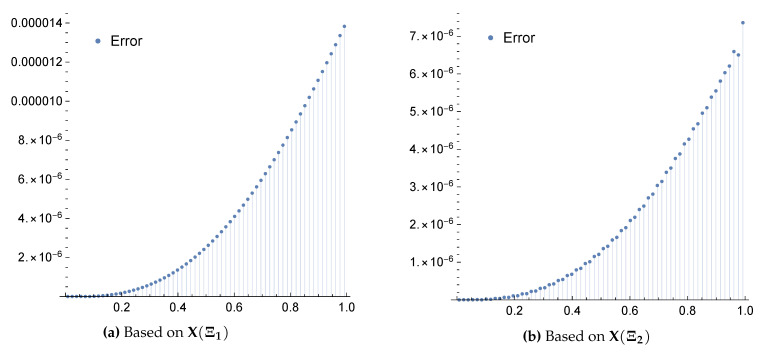
Error plots for Example 4 when m=4 using the framelet systems $X(\Xi_{1})$ and $X(\Xi_{2})$.

*The exact solution for this equation is u(x)=x. Again, applying the proposed algorithm yields the numerical results presented in Table 3 and Table 4 and the graphical illustration for comparison of exact, approximate and error results in Figure 5 and Figure 6.*

**Example 5.**  *Consider the following FVIE.*

(9) $$\left\{\begin{array}{c} D^{\sqrt{3}}u\left(x\right){-}_{0}^{x}(\sin(t)\sin(x)+\cos(t)\cos(x))u(t)dt=\frac{2\left(\sqrt{3}+2\right)}{\Gamma \left(2-\sqrt{3}\right)}x^{2-\sqrt{3}}+2\left(x-\sin\left(x\right)\right),\\ u(0)=0\\ u^{\prime }\left(0\right)=1. \end{array}\right.$$

*Note that Equation (9) can be reduced to*

$$\left\{\begin{array}{c} {\frac{1}{\Gamma \left(2-\sqrt{3}\right)}}_{0}^{x}\frac{u^{\prime \prime }\left(t\right)}{{\left(x-t\right)}^{\sqrt{3}-1}}dt{-}_{0}^{x}(\sin(t)\sin(x)+\cos(t)\cos(x))u(t)dt=\frac{2\left(\sqrt{3}+2\right)}{\Gamma \left(2-\sqrt{3}\right)}x^{2-\sqrt{3}}+2\left(x-\sin\left(x\right)\right),\\ u(0)=0\\ u^{\prime }\left(0\right)=1. \end{array}\right.$$

**Figure 7:**
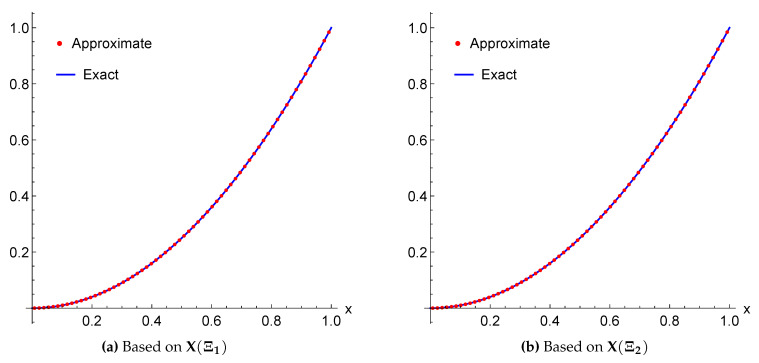
A Comparison between the exact and approximate solutions of Example 5 for m=3 using the framelet systems $X(\Xi_{1})$ and $X(\Xi_{2})$.

**Figure 8:**
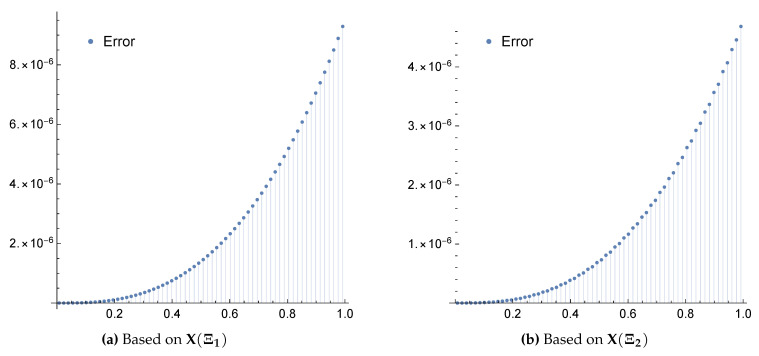
Error plots for Example 4 using the framelet systems $X(\Xi_{1})$ and $X(\Xi_{2})$ when m=4.

**Figure 9:**
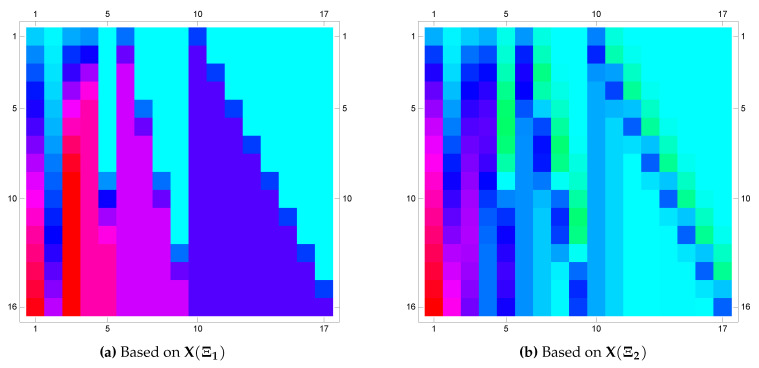
Matrix plot of the coefficients of the approximate solution $E_{2}u$ of Example 5 based on the framelet systems $X(\Xi_{1})$ and $X(\Xi_{2})$.

*The exact solution for this equation is u(x)=x. Again, applying the proposed algorithm yields the numerical results presented in Table 5 and the graphical illustration for the comparison of exact, approximate and error results in Figure 7 and Figure 8. We also provide the matrix plot of the coefficients of the approximate solution based on different framelet systems and when m=2. The matrix plots are depicted in Figure 9.*

**Example 6.**  *Consider the following FVIE.*

(10) $$\left\{\begin{array}{c} D^{1/3}u\left(x\right){-}_{0}^{x}\left(xt+x^{2}t^{2}\right)u\left(t\right)dt=\frac{\sqrt{\pi }\sqrt[{6}]{x}}{2\Gamma \left(\frac{7}{6}\right)}-\frac{2}{35}x^{7/2}\left(5x^{2}+7\right),\\ u(0)=0. \end{array}\right.$$

*Note that Equation (10) can be reduced to*

$$\left\{\begin{array}{c} {\frac{1}{\Gamma \left(2/3\right)}}_{0}^{x}\frac{u^{\prime }\left(t\right)}{{\left(x-t\right)}^{1/3}}dt{-}_{0}^{x}\left(xt+x^{2}t^{2}\right)u\left(t\right)dt=\frac{\sqrt{\pi }\sqrt[{6}]{x}}{2\Gamma \left(\frac{7}{6}\right)}-\frac{2}{35}x^{7/2}\left(5x^{2}+7\right),\\ u(0)=0. \end{array}\right.$$

**Figure 10:**
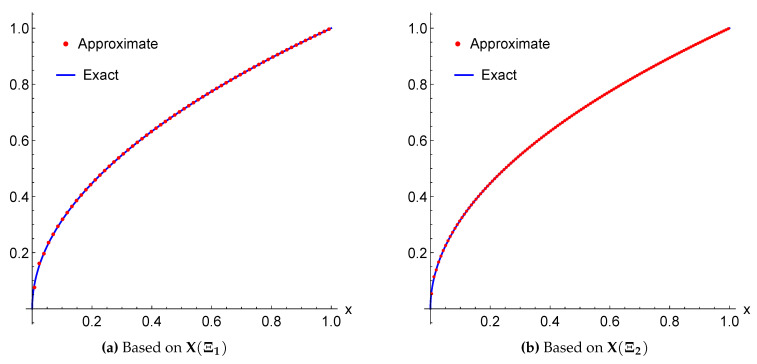
A Comparison between the exact and approximate solutions of Example 6 for m=4 using the framelet systems $X(\Xi_{1})$ and $X(\Xi_{2})$.

*The exact solution for this equation is $u\left(x\right)=\sqrt{x}.$ Again, applying the proposed algorithm yields the numerical results presented in Table 6 and the graphical illustration for the comparison of exact and approximate solutions in Figure 10.*

## 4. Conclusions

The framelet system we used in this paper was generated using three wavelet frame functions with compact support and constructed based on using the non-negative functions, B-splines.

We have also established two important results on the existence and uniqueness of the Equations (1) and (2) considered in this paper. The proposed method was tested by numerically solving many important examples of fractional Volterra integral equations. This work is an extension of the work published in [47] by involving the fractional order derivative, namely, the Caputo fractional derivative sense.

The approximate solutions are supported by numerical evidence given in Table 1, Table 2, Table 3, Table 4, Table 5 and Table 6, and graphical illustrations in Figure 3, Figure 4, Figure 5, Figure 6, Figure 7, Figure 8, Figure 9 and Figure 10, wherein excellent agreement with the exact solutions was accomplished with only a few framelet truncated partial sums.

Based on the graphical and numerical evidence, we conclude that the accuracy of the method is increased by two important factors:

1. Number of terms of the partial sum of the framelet truncated expansion being used;
2. The vanishing moments order of the framelet system being used, where increasing these terms will result an increase in the accuracy as well as the efficiency of the algorithm.

## Figures and Tables

**Table 1:** Numerical results of Example 3 using the framelet systems $X(\Xi_{1})$ and $X(\Xi_{2})$ for m=3.

*x*	*Exact*	$E_{m}u$ via $X(\Xi_{1})$	$E_{m}u$ via $X(\Xi_{2})$
0.1	0.01	7.26467 × $10^{-4}$	1.85352 × $10^{-5}$
0.2	0.04	9.10220 × $10^{-4}$	6.91144 × $10^{-5}$
0.3	0.09	1.82277 × $10^{-4}$	1.51510 × $10^{-4}$
0.4	0.16	3.51120 × $10^{-3}$	2.67941 × $10^{-4}$
0.5	0.25	4.47280 × $10^{-3}$	4.28731 × $10^{-4}$
0.6	0.36	8.36896 × $10^{-3}$	7.18203 × $10^{-3}$
0.7	0.49	1.17160 × $10^{-3}$	1.06917 × $10^{-3}$
0.8	0.64	1.64979 × $10^{-2}$	1.51795 × $10^{-2}$
0.9	0.81	2.29604 × $10^{-2}$	2.08445 × $10^{-2}$
1.0	1.00	2.99714 × $10^{-2}$	2.72612 × $10^{-2}$

**Table 2:** Numerical results of Example 3 using the framelet systems $X(\Xi_{1})$ and $X(\Xi_{2})$ for m=4.

*x*	*Exact*	$E_{m}u$ via $X(\Xi_{1})$	$E_{m}u$ via $X(\Xi_{2})$
0.1	0.01	3.64554 × $10^{-6}$	1.73553 × $10^{-6}$
0.2	0.04	5.91254 × $10^{-5}$	2.96533 × $10^{-6}$
0.3	0.09	4.66455 × $10^{-5}$	1.09373 × $10^{-5}$
0.4	0.16	2.51037 × $10^{-5}$	2.35522 × $10^{-5}$
0.5	0.25	2.46092 × $10^{-5}$	4.22966 × $10^{-4}$
0.6	0.36	4.63545 × $10^{-4}$	7.11944 × $10^{-4}$
0.7	0.49	1.30932 × $10^{-3}$	1.55358 × $10^{-4}$
0.8	0.64	2.35355 × $10^{-3}$	1.24774 × $10^{-4}$
0.9	0.81	1.38865 × $10^{-3}$	1.04342 × $10^{-3}$
1.0	1.00	3.53446 × $10^{-3}$	3.39882 × $10^{-3}$

**Table 3:** Numerical results of Example 4 using the framelet systems $X(\Xi_{1})$ and $X(\Xi_{2})$ for m=3.

*x*	*Exact*	$E_{m}u$ via $X(\Xi_{1})$	$E_{m}u$ via $X(\Xi_{2})$
0.1	0.01	7.88293 × $10^{-8}$	2.92921 × $10^{-9}$
0.2	0.04	7.67236 × $10^{-7}$	1.36504 × $10^{-8}$
0.3	0.09	2.36706 × $10^{-6}$	1.05287 × $10^{-7}$
0.4	0.16	1.09108 × $10^{-5}$	2.91769 × $10^{-6}$
0.5	0.25	1.27581 × $10^{-5}$	4.42031 × $10^{-6}$
0.6	0.36	1.69445 × $10^{-5}$	5.90872 × $10^{-6}$
0.7	0.49	2.44359 × $10^{-5}$	1.06485 × $10^{-5}$
0.8	0.64	3.32952 × $10^{-5}$	1.27942 × $10^{-5}$
0.9	0.81	4.33451 × $10^{-5}$	2.18122 × $10^{-5}$
1.0	1.00	5.43243 × $10^{-5}$	2.98557 × $10^{-5}$

**Table 4:** Numerical results of Example 4 using the framelet systems $X(\Xi_{1})$ and $X(\Xi_{2})$ for m=4.

*x*	*Exact*	$E_{m}u$ via $X(\Xi_{1})$	$E_{m}u$ via $X(\Xi_{2})$
0.1	0.01	1.31612 × $10^{-8}$	1.91626 × $10^{-9}$
0.2	0.04	51.7061 × $10^{-7}$	1.45913 × $10^{-9}$
0.3	0.09	6.39942 × $10^{-7}$	1.06851 × $10^{-8}$
0.4	0.16	0.41406 × $10^{-6}$	3.21467 × $10^{-7}$
0.5	0.25	0.49218 × $10^{-6}$	6.80432 × $10^{-7}$
0.6	0.36	0.60156 × $10^{-6}$	1.20816 × $10^{-6}$
0.7	0.49	0.69531 × $10^{-6}$	3.03948 × $10^{-6}$
0.8	0.64	8.53345 × $10^{-6}$	4.26697 × $10^{-6}$
0.9	0.81	1.10689 × $10^{-5}$	5.81013 × $10^{-6}$
1.0	1.00	1.38297 × $10^{-5}$	7.36015 × $10^{-6}$

**Table 5:** Numerical results of Example 5 using the framelet systems $X(\Xi_{1})$ and $X(\Xi_{2})$ for m=4.

*x*	*Exact*	$E_{m}u$ via $X(\Xi_{1})$	$E_{m}u$ via $X(\Xi_{2})$
0.1	0.01	1.62034 × $10^{-8}$	8.34395 × $10^{-9}$
0.2	0.04	1.03814 × $10^{-7}$	5.23015 × $10^{-8}$
0.3	0.09	3.56716 × $10^{-7}$	1.85430 × $10^{-7}$
0.4	0.16	7.48135 × $10^{-7}$	3.84891 × $10^{-7}$
0.5	0.25	1.45995 × $10^{-6}$	6.85008 × $10^{-7}$
0.6	0.36	2.32864 × $10^{-6}$	1.10671 × $10^{-6}$
0.7	0.49	3.47190 × $10^{-6}$	1.73964 × $10^{-6}$
0.8	0.64	5.19781 × $10^{-6}$	2.46654 × $10^{-6}$
0.9	0.81	7.05227 × $10^{-6}$	3.56779 × $10^{-6}$
1.0	1.00	9.28981 × $10^{-6}$	4.45749 × $10^{-6}$

**Table 6:** Numerical results of Example 6 using the framelet systems $X(\Xi_{1})$ and $X(\Xi_{2})$ for m=4.

*x*	*Exact*	$E_{m}u$ via $X(\Xi_{1})$	$E_{m}u$ via $X(\Xi_{2})$
0.1	0.01	1.65534 × $10^{-6}$	3.23863 × $10^{-7}$
0.2	0.04	2.34587 × $10^{-6}$	5.74663 × $10^{-7}$
0.3	0.09	2.63882 × $10^{-7}$	0.64773 × $10^{-8}$
0.4	0.16	8.38292 × $10^{-7}$	1.33748 × $10^{-8}$
0.5	0.25	6.37474 × $10^{-7}$	2.92292 × $10^{-8}$
0.6	0.36	7.38381 × $10^{-6}$	7.35377 × $10^{-7}$
0.7	0.49	1.22234 × $10^{-6}$	4.43444 × $10^{-7}$
0.8	0.64	4.10292 × $10^{-6}$	1.92556 × $10^{-6}$
0.9	0.81	5.37333 × $10^{-5}$	2.01111 × $10^{-5}$
1.0	1.00	2.32444 × $10^{-5}$	2.22298 × $10^{-5}$
